# Social Bullying Among Undergraduates: The Roles of Internet Gaming Disorder, Risk-Taking Behavior, and Internet Addiction

**DOI:** 10.3389/fpsyg.2022.830794

**Published:** 2022-07-13

**Authors:** Chinonso L. Nwanosike, Ikechukwu V. N. Ujoatuonu, Gabriel C. Kanu, Obinna O. Ike, Tochukwu J. Okeke

**Affiliations:** Department of Psychology, Faculty of the Social Sciences, University of Nigeria, Nsukka, Nigeria

**Keywords:** internet addiction, internet gaming disorder (IGD), risk-taking behavior, social bullying, undergraduate students

## Abstract

An issue that affects the academic engagement, performance, health and wellbeing of university undergraduates is bullying. Substantial literature has examined the predictors of bullying perpetration, but there is little research on the contributions of internet-related factors and the propensity to take risks in bullying. We examined the roles of IGD, risk-taking behavior, and internet addiction in social bullying. Four instruments were used for data collection, namely: Young Adult Social Behavior Scale (YASB), the Internet Gaming Disorder Scale (IGDS9-SF), Domain-Specific Risk-Taking Scale, and the Internet Addiction Test (IAT) Scale. Participants were 552 undergraduate students from the University of Nigeria, Nsukka consisting of 143 males and 409 females (age range = 17–32 years; *M* = 21.45; *SD* = 2.71). Results of regression analysis showed that gaming disorder (GD) and risk-taking behavior had positive associations with social bullying. Thus, the more people grow addicted to internet gaming and takes more risks, the more they are likely to become bullies. Internet addiction had no significant association with social bullying. Efforts should be made to minimize the rate of dysfunctional internet use, GD and risk-taking behaviors of undergraduates in order to curtail bullying perpetration.

## Introduction

Bullying is common in schools (Hanani, 2021). Bullied students may experience anxiety, despair, suicidal ideation, low energy, and sleep deprivation, affecting their mood, focus, overall wellbeing, and internet addiction for bullied victims at school (Saldıraner and Gızır, 2021). According to Menesini and Salmivalli (2017), bullying occurs even among classmates, peers and significant others. Early studies (e.g., Scheithauer et al., 2006; Omoniyi, 2013) focused primarily on direct bullying, such as physical and verbal abuse. Recently, more research interests have been turned to indirect forms of bullying like social bullying (Dailey et al., 2015). Like social bullying, this indirect form of bullying appears to be a natural and widespread aspect of growing up in developing and underdeveloped nations like Nigeria (Omoniyi, 2013). It has substantial consequences for children’s socio-emotional development, anxiety, social disengagement, and depression. Parents who view social bullying as a regular part of growing up may fail to provide emotional and instrumental support to their children because of a lack of family and school programs, practices and policies (Rigby, 2013). Also, they may fail to intervene to prevent further peer bullying (Omoniyi, 2013).

Social bullies may want to control others, act maliciously, have poor social skills and have difficulty getting along with others (Hanani, 2021). They perceive a power imbalance between themselves and their victims, lack empathy with social intelligence, are often insecure and bully others into making themselves feel better, and subject their victims to recurrent discomfort, provocation, and retaliation due to their behavior (Cho and Lee, 2018). Social or relational bullying entails purposefully harming people’s reputations or relationships (Dailey et al., 2015). Social bullying is defined by Crothers et al. (2019) as social behaviors that threaten and trigger loss of friendship or connection through isolation or alienation and the potential for bullies to cause harm to victims by exploiting a relationship. It includes purposely leaving someone out, advising other children not to be friends with someone, spreading rumors against someone, and publicly humiliating someone (Scheithauer et al., 2006). Social bullying can occur at secondary schools, on campus, or outside secondary schools and universities (Saldıraner and Gızır, 2021). However, it is most widespread in schools due to interactions (Rigby, 2013).

Often, young people bully without acknowledging it, while others are bullied without realizing it (Arcadepani et al., 2019). Constant and systematic misappropriation of an authority constitutes this type of act (Saldıraner and Gızır, 2021). Slapping, name-calling, rumor, exclusion from groups, and even social media harassment/embarrassment are all forms of social bullying (Cho and Lee, 2018). When social-related violence and bullying occur, Students’ and children’s rights to education, health, and wellbeing are violated (Lee et al., 2018). Also, Students’ academic performance, career development, mental health, and quality of life are negatively impacted by social bullying (Omoniyi, 2013). School intruding ideas (such as spontaneous recollections of a time when they were bullied), absences, distress, apathy, or a lack of enthusiasm, desensitization, and dropping out of formal education after high school are also more common among undergraduates that experience social bullying (Menesini and Salmivalli, 2017). These students may turn to internet addiction (Boniel-Nissim and Sasson, 2018) and risk-taking behavior (Chang et al., 2015) to avert anxiety, despair and suicidal ideation (Andreassen and Pallesen, 2014). Despite the dangers that school bullying poses to Students’ academic performance and general health, little research on this issue has been done in Nigeria. Our primary goal in this study was to examine the roles of IGD, risk-taking behavior, and internet addiction in social bullying among Nigerian undergraduate students. There is an increase in criminal acts and social bullying in schools, leading to some kidnappings and deaths among pupils (Hassan-Wuyo, 2022; Tauna, 2022); our study is relevant to the Nigerian environment at this point. We aim to see how this study can help school administrators for policies that could curb this menace. Our first independent variable is IGD.

One of the most common online hobbies among undergraduates is internet gaming, which interferes with their psychological (mental and emotional) wellbeing, academic, career, social connections (Ozcinar, 2011), relationships with significant others, and daily functioning (Cinar et al., 2017). Internet gaming disorder (IGD), also known as GD or online gaming problem (OGP), is defined by Macur and Pontes (2021) as regular and recurring participation in video games that significantly impact everyday life and employment and education. Also, Pontes and Griffiths (2015) define IGD as an insatiable desire to play online games to addiction, which can manifest as compulsive behavior. IGD is caused by addiction, excessive use, and time spent playing games (Statista, 2021). Problem gambling, sadness, social disengagement, depression, somatization, insomnia, mood disorders, obesity, and anxiety disorders are all symptoms of IGD, and they can all be exacerbated by the condition (Jeong et al., 2020).

IGD can lead to a loss of self-control, a myriad of health issues, and problems in personal relationships (Shin et al., 2020), leading to social bullying if the focus shifts from enjoyment to obsession. Undergraduates’ real-life relationships and aspects of their personal, professional, mental health, and social lives may be harmed by internet gaming problems and habits (Ozcinar, 2011). Although internet gaming is often regarded as a typical pastime for adolescent and adult males worldwide, there is growing concern that many gamers are becoming addicted (Wang et al., 2019), which may trigger social bullying. According to researchers (e.g., Ozcinar, 2011; Andreassen and Pallesen, 2014; VondráčKová and Gabrhelík, 2016; Cinar et al., 2017; Statista, 2021), people are addicted to the internet in general or to specific online activities like online gambling, gaming, or cybersex. In this case, internet gamers might channel their rage toward social bullying to substitute for emotionally toxic characters in their lives. Aggression, mood, self-esteem, and impulsivity induced by online gaming can harm undergraduates’ real-life relationships and possibly trigger social bullying (Zsila et al., 2017).

There has been no research on IGD and social bullying yet. Although studies (e.g., Wang et al., 2019; Jeong et al., 2020; Shin et al., 2020; Macur and Pontes, 2021) have found a significant relationship between IGD and impairment in everyday activities, as well as a relationship with health-related quality of life, profiles and associated risk factors, the neural mechanism of the relationship between impulsivity and emotion dysregulation with IGD patients, and reduced loss aversion and inhibitory control in adolescents. Based on these findings, we hypothesized that IGD and social bullying might be linked. Although most of these researches were conducted in western cultures, there is a need to understand the relationship between IGD and social bullying in Africa.

Similarly, social bullying may be associated with risk-taking behavior. However, the association between risk-taking and social bullying is complicated (Scheithauer et al., 2006). Bullies are associated with overestimation in taking-risk perception, whereas the victim was associated with underestimation (Poon, 2016). As a result, risk-taking is linked to victims who undervalue themselves and bullies who overestimate themselves. High risk-taking modulation and impulsive decision-making have been linked to both bullies and victims (Blais and Weber, 2006). Victims of bullying frequently undervalue both the positive and negative aspects of their situation. Socially bullied kids may engage in risk-taking behavior to cope with victimization-related stress (Cho and Lee, 2018). When it comes to the conduct of young people, it is common to hear about their penchant for risk-taking and irresponsible community behavior. According to researchers (e.g., Blais and Weber, 2006; Scheithauer et al., 2006; Poon, 2016; Hanani, 2021), undergraduates perceive bullying as a risk-taking behavior that they must engage in, similar to drinking, smoking, engaging in unprotected sex, and using drugs to intimidate others.

Lee et al. (2018) defined risk-taking as any mindfully or unknowingly controlled conduct with a speculative risk about the results and the possible advantages and disadvantages of an individual’s physical, fiscal, and social wellbeing or the wellbeing of others. According to Boniel-Nissim and Sasson (2018), increased reward-seeking behavior among students, particularly among their peers, is a powerful motivator for risk-taking behavior. The dopaminergic system in Students’ brains is rewired during their academic careers, which accounts for a large part of their proclivity to take risks (Kritsotakis et al., 2017) and engage in social bullying.

Individuals engage in risk-taking behavior in high-payoff acts with immediate positive or adverse consequences rather than long-term costs (Crothers et al., 2019). The more impulsive a victim is, the more likely they will endanger themselves (Hanani, 2021). People of all ages are affected by emotions and impulses, so risk-taking tendencies increase with age (Crothers et al., 2019). There is a lack of empirical research on the possible association between risk-taking behavior and social bullying among Nigerian undergraduate students. However, Tauna (2022) stated that cultism, kidnapping, brutality, and death are risk-taking behavior practiced by college and undergraduate students that might influence social bullying in Nigeria.

Another purpose of the present study was to examine the associations of IGD, risk-taking behavior, social bullying, and the moderator role of internet addiction. Studies (e.g., Li et al., 2019; Lin et al., 2020) suggest that internet addiction could be a moderator of bullying, peer and cyber victimization, depressive and anxiety symptoms, with psychological and physical symptoms. On that note, the researchers decided to study internet addiction as a moderator. According to Geirdal et al. (2021), social media and internet addiction contribute to an increase in bullying, which affects around 40% of the population at some point in their lives. Young (1996) defined internet addiction as a compulsive urge and resorting to spending a lot of time online for emotional support, affecting victims or addicts, life engagements (academic, business, or employment), neurological (health), psychological problems (substance dependence) and social and interpersonal issues (sextortion and gambling addictions, bullying, and revenge porn). Also, VondráčKová and Gabrhelík (2016) defined internet addiction as an insatiable desire to spend a significant amount of time on the internet, harming one’s relationships, productivity, overall wellbeing, and others.

According to Brighi et al. (2012), the rise of social media platforms such as Facebook, WhatsApp, Instagram, and other internet media-based platforms has resulted in increased bullying at schools, as well as lower academic engagement and performance, and increased symptoms of poor mental health when compared to peers who do not use social media platforms like Facebook, WhatsApp, and Instagram. Unwelcome social comparisons, risk-taking behavior, and internet addiction, which encourage a preoccupation with things other than individual achievement (Cinar et al., 2017), might be the root of social bullying and other negative consequences on academic engagement, performance, and career development.

There has been no research on moderating the role of internet addiction in the associations of IGD, risk-taking behavior, and social bullying yet. Although studies (e.g., Ko et al., 2012; Andreassen et al., 2017; Alexandraki et al., 2018; Boniel-Nissim and Sasson, 2018; Kircaburun and Griffiths, 2018; Kircaburun et al., 2018; Kırcaburun et al., 2019; Li et al., 2019; Simsek et al., 2019; Boer et al., 2021; Çimke and Cerit, 2021) have found a significant relationship between adolescents’ internet addiction, social media use intensity and addiction, cyberbullying and victimization, psychiatric disorder, social media use problems, mental health, depressive and anxiety symptoms, school functioning and peer victimization. Also, internet addiction has been found to have a significant association between harsh parenting, maltreatment and bullying (Lo et al., 2021), child abuse, social phobia, depression and bullying victimization (Malaeb et al., 2020), parental mediation, cyberbullying and depression (Chang et al., 2015). Based on these findings, we hypothesized that internet addiction and social bullying might be linked. Although most of these researches were conducted in western cultures, there is a need to understand the relationship between internet addiction and social bullying in Africa.

Our study is anchored on the use and gratification theory by Katz et al. (1973) that can determine undergraduates’ motivations (e.g., risk-taking), media consumption (e.g., internet gaming and addiction) and satisfaction (social bullying). The theory assumes that social, educational, economic, political environment and psychological factors influence media and internet use, risk-taking behavior and social bullying. According to the theory of uses and gratification, people use the internet and media by taking risks for two types of gratification: seeking and receiving. It focuses on people’s motivations for using social media and how they use it. The uses and gratification theory explained how social media users could control their media consumption, integrate it into their lives, and gain satisfaction (Musa et al., 2015). On that note, we hypothesized that:

1. IGD will be significantly associated with social bullying among undergraduates.
2. Risk-taking behavior will be significantly associated with social bullying among undergraduates.
3. Internet addiction will be significantly associated with social bullying among undergraduates.
4. Internet addiction will moderate the relationship between IGD and social bullying among undergraduates.
5. Internet addiction will moderate the relationship between risk-taking behavior and social bullying among undergraduates.

## Materials and Methods

### Participants

Participants were 552 undergraduates (male = 143; female = 409) with age ranging from 17 to 32 years (*M* = 21.45, *SD* = 2.71). They were drawn from the University of Nigeria, Nsukka. Five hundred thirty-six (97.1%) of the participants were single, 15 (2.7%) were married, and 1 (0.2%) were divorced. In terms of ethnicity, 446 (80.8%) were Igbo, 80 (14.5%) were Hausa, and 26 (4.7%) were Yoruba. By religion, 462 (83.7%) were Christians, 74 (13.4%) were Muslims, 11 (2.0%) were African traditionalists, and 3 (0.5%) belonged to other religious groups. On the level of study, 147 (26.6%) were in the first year, 248 (44.9%) were in the second year, 125 (22.6%) were in the third year, 29 (5.3%) were in the fourth year, and 3 (0.5%) were in the fifth year.

### Instruments

We used four measures in the study: The YASB, the Internet Gaming Disorder Scale (IGDS9-SF), the Domain-Specific Risk-Taking Scale, and the Internet Addiction Test.

### Young Adult Social Behavior Scale

The Young Adult Social Behavior Scale (YASBS), a 14-item measure, was developed by Crothers et al. (2008) as an alternative to existing measures of relational aggression to assess the contributions of direct relational aggression and social aggression the larger concept of relational or social bullying. This instrument measures self-reported relational and social aggression with indirect aggression and behaviors of interpersonal maturity primarily represent the same construct in adolescents and young adults. Each item on the scale was measured using a 5-point Likert-type scale ranging from “Never” to “Always.” Some of the items are: “When I do not like someone’s personality, I derive a certain degree of pleasure when a friend listens to and agrees to my assessment of the person’s personality,” “I break-a friend’s confidentiality to have a good story to tell.” “I intentionally exclude friends from activities to make a point with them.” Higher scores represented higher social bullying. Tucker-Lewis index of 0.97 and a comparative fit index of 0.98 were used to provide strong statistical support for the scale (Crothers et al., 2008). Crothers et al. (2008) reported a reliability of 0.80. For the present study, we obtained a Cronbach’s alpha of 0.78.

### Internet Gaming Disorder Scale–Short-Form

Internet Gaming Disorder Scale is a 9-item measure developed by Pontes and Griffiths (2015) to measure IGD. Each item of the scale was scored on a 5-point Likert-type scale ranging from “Never” (1) to “Very often” (5). Some of the items are: “Do you feel preoccupied with your gaming behavior? “Have you jeopardized or lost an important relationship, job or an educational or career opportunity because of your gaming activity?” Total scores can be obtained by summing up all responses given to all nine items of the IGDS9-SF and can range from a minimum of nine to a maximum of forty-five points, with higher scores indicative of a higher degree of IGD. Pontes and Griffiths (2015) obtained a reliability coefficient of 0.96. To obtain concurrent validity, Ling et al. (2021) correlated the items of the Online Gaming Questionnaire-Short Form with IGDS and a positive correlation was obtained (*r* = 0.78, *p* < 0.05). We obtained a Cronbach’s alpha of 0.87 in the present study.

### Domain-Specific Risk-Taking (Adult) Scale

Domain-Specific Risk-Taking (Adult) Scale (DOSPERT) is a 30-item measure developed by Blais and Weber (2006) to assess the likelihood with which respondents might engage in risky activities/behaviors. It has five domains (i.e., ethical, financial, health/safety, social, and recreational risks) and on a seven-point Likert scale format ranging from 1 (Extremely Unlikely) to 7 (Extremely Likely). Sample items include “Having an affair with a married man/woman” (Ethical), “Investing 10% of your annual income in a new business venture” (Financial), “Engaging in unprotected sex” (Health/Safety), “Disagreeing with an authority figure on a major issue” (Social), and “Taking a weekend sky-diving class” (Recreational). Item ratings are added across all items of a given subscale to obtain subscale scores. Higher scores indicate greater risk-taking in the domain of the subscale. Blais and Weber (2006) reported a coefficient alpha ranged from 0.70 to 0.84 (mean α = 0.78). In the present study, a global score was used. In the present study, Cronbach’s alpha of 0.89 was obtained.

### Internet Addiction Test

Internet Addiction Test (IAT) Questionnaire is a 20-item scale that measures addictive internet use developed by Young (1996). It has six dimensions: salience, excessive use, neglect of work, anticipation, lack of control, and neglect of social life. It is measured on a six-Likert-scale format ranging from 0 (Does not apply) to 5 (Always). Some of the items include: “How often do you fear that life without the internet would be boring, empty, and joyless?,” “How often do you lose sleep due to late-night log-ins? Young (1996) reported a Cronbach’s alpha that ranged from 0.54 to 0.82. We obtained a Cronbach’s alpha of 0.78 for the present study.

### Procedure

The research was carried out at the University of Nigeria, Nsukka. We used a simple random sampling method to select and collect data from three faculties: Biological Sciences, Law, and Social Sciences. One out of the two simple random sampling methods, we used the lottery method. Because the researchers do not need to have prior technical knowledge about their vast population size, the lottery method will reduce bias and make it easy to pick a sample size from the larger, well-informed population to harvest a high-quality data set. The psychology research ethics committee of the University of Nigeria, Nsukka, approved the study. The undergraduates were asked to participate in a survey on social bullying, IGD, risk-taking and internet addiction. Those who agreed to participate in the research were given a questionnaire to fill out. Minimum age of 17 years was required, and the ability to read and understand the English language. Participants were encouraged to be as “truthful” and “sincerely” as possible in their responses to each questionnaire. However, participants were informed that they were free to withdraw at any time during the study with no repercussions. The questionnaire was then distributed to those who agreed to participate in the study at their libraries and after morning lectures. We verbally expressed gratitude to the participants, and depending on their preference; we thanked and appreciated the participants with either a blue or black pen. Five hundred and eighty questionnaires were distributed at random. Five hundred and fifty-two questionnaires were valid for computation in the statistical package, with more than 85% return rate.

### Design/Statistics

The Hayes regression-based PROCESS macro for SPSS was used to analyze the data collected for the research. The robust PROCESS macro for SPSS is favored over ordinary regression analysis (Hayes, 2013). PROCESS performs regression-based path analysis and produces product terms to investigate interaction effects, centering predictor variables automatically before analysis. The Hayes PROCESS is currently the most widely accepted approach for moderation tests (see Onyedire et al., 2019; Li et al., 2019; Lin et al., 2020). If a product term (that is, the interaction of predictor and moderator) was significant, it meant that in the presence of the moderator(s), the link between the relationship variable (e.g., internet addition) and the criterion variable (social bullying) was either stronger or weaker (e.g., internet gaming disorder and risk-taking behavior).

## Results

In Table 1, the range, mean, and standard deviations of age, internet addiction, internet game disorder, risk-taking behaviour and social bullying were shown and tabulated.

**TABLE 1:** Descriptive statistics for the study variables.

Variables	Range	Mean	*SD*
Age	17–32	21.45	2.71
Internet addiction	7–92	42.40	16.90
Internet game disorder	9–75	21.60	8.63
Risk taking	36–187	102.86	26.55
Social bullying	5–25	10.17	3.89

In Table 2, male were older in age, and were involved in social bullying. Older age was associated with higher level of study, and internet addiction. IGD was positively associated with risk taking.

**TABLE 2:** Correlations of demographic variables, internet addiction, internet gaming disorder, risk-taking, and social bullying.

Variables		1	2	3	4	5	6
1	Gender	−					
2	Age	−0.09*	–				
3	Level of study	−0.03	0.09*	−			
4	Internet addiction	0.03	0.14**	−0.01	−		
5	Internet gaming disorder	0.02	−0.01	−0.02	0.08	−	
6	Risk taking	0.01	0.02	−0.05	0.00	0.30***	−
7	Social bullying	0.14**	0.01	−0.01	0.05	0.31***	0.18***

*p < 0.05; **p < 0.01; ***p < 0.001; Gender (0 = Male; 1 = female).

In Table 3, GD was significantly positively associated with social bullying (*B* = 0.14, *p* = 0.009). For every one unit rise in GD, social bullying increases by 0.14 units. Internet addiction was not significantly associated with social bullying (*B* = 0.01). The interaction of GD and internet addiction was not significant (*B* = 0.00), indicating that internet addiction did not moderate the relationship between GD and social bullying. The model explained about 12% of the variance in social bullying, *R*^2^ = 0.12, and was significant, *F*(4, 547) = 17.31, *p* = 0.000.

**TABLE 3:** Hayes PROCESS macro results for internet gaming disorder predicting social bullying with internet addiction as moderator.

Variables	*B*	*t*	*p*-level	95%CI
Gender	1.18	3.31	0.001	[0.48, 1.89]
Gaming disorder (GD)	0.14	2.61	0.009	[0.03, 0.24]
Internet addiction (IA)	0.01	0.22	0.825	[−0.05, 0.06]
GD × IA	0.00	−0.01	0.990	[−0.00, 0.00]

In Table 4, risk taking was significantly positively associated with social bullying (*B* = 0.04, *p* = 0.020). For every one unit rise in risk taking, social bullying increases by 0.04 units. Internet addiction was not significantly associated with social bullying (*B* = 0.04). The interaction of risk taking and internet addiction was not significant (*B* = −0.00), indicating that internet addiction did not moderate the relationship between risk taking and social bullying. The model explained about 6% of the variance in social bullying, *R*^2^ = 0.06, and was significant, *F*(4, 547) = 7.96, *p* = 0.000.

**TABLE 4:** Hayes PROCESS macro results for risk taking predicting social bullying with internet addiction as moderator.

Variables	*B*	*t*	*p*-level	95%*CI*
Gender	1.21	3.27	0.001	[0.48, 1.93]
Risk taking (RT)	0.04	2.32	0.020	[0.01, 0.07]
Internet addiction (IA)	0.04	1.04	0.438	[−0.00, 0.00]
RT × IA	−0.00	−0.78	0.014	[0.01, 0.09]

## Discussion

The primary goal of our study was to examine the moderating role of internet addiction in the relationship between IGD, risk-taking behavior and social bullying among undergraduates. The result showed that IGD was positively associated with social bullying. Thus, hypothesis one was confirmed. Our study result is consistent with the findings from studies (e.g., Andreassen and Pallesen, 2014; Adachi and Willoughby, 2016; Baldry et al., 2016; Cinar et al., 2017; Lee et al., 2017; Kircaburun et al., 2018; Li et al., 2019; Lin et al., 2020) who found that internet gaming behavior, the topology of video GD, problematic video game addiction, poor relationship with parents, competitive video gameplay and cyberbullying to have significant associations with risk factors of recurrent online activity and compulsive use of video games, psychopathology (social bullying), sadness, suicide, and pattern of disruptive behavior (social bullying), violence and aggression among adolescents and young adults. Also, our study finding has confirmed Tauna’s (2022) assertion that adolescents in Nigeria engage in criminal acts like social bullying, murder, kidnappings, and cultism in Nigerian schools due to increased internet gaming and gambling and other factors related to negative internet experiences. This finding showed that constantly engaging in internet gaming for pleasure may increase the tendency to perpetuate social bullying.

Getting pleasure from internet gaming could jeopardize the need to want to connect with others which becomes distressing (Ling et al., 2021). The reason is that those who get addicted to internet gaming for fun or escape have developed a new sense of attachment (Jeong et al., 2020), and that is why they could care less about others and, thus, the increasing tendency to bully (Fitzpatrick and Bussey, 2018). Also, internet gambling disorder, if left unchecked, internet can lead to social anxiety, bullying, disengagement, distinct emotional disturbance, and possibly suicide. Policies and programs addressing what families, schools, and significant others can do to create anti-bullying initiatives that can minimize the occurrence of social bullying in schools should be implemented.

Furthermore, our findings demonstrated that taking risks was significantly associated with social bullying among undergraduates, confirming the second hypothesis. This finding is in agreement with previous findings (e.g., Kritsotakis et al., 2017; Zsila et al., 2017; Arcadepani et al., 2019; Wang et al., 2019) who stated that substance use and sexual risk-taking behaviors had been linked to an increased likelihood of physical, verbal, and social bullying. Students who take risks are more likely to engage in social bullying. Those who engage in high-risk behavior are more likely to injure others socially because they believe they need to boost their self-esteem and, as a result, believe that social bullying is acceptable. A link exists between social bullying and risk-taking behavior. Also, our study has confirmed Hassan-Wuyo’s (2022) assertion that adolescents in Nigeria engage in kidnapping, cultism, and other risk-taking behavior that might influence or are linked to social bullying within and outside school environments.

The result showed that internet addiction was not significantly associated with social bullying, and thus, the third hypothesis was rejected. That is to say that internet addiction does not strengthen or lessen the extent to which people engage in social bullying. This finding contradicts previous findings by Ko et al. (2012); Andreassen et al. (2017); Alexandraki et al. (2018); Boniel-Nissim and Sasson (2018); Kircaburun and Griffiths (2018), Kircaburun et al. (2018), Kırcaburun et al. (2019), Li et al. (2019); Simsek et al. (2019), Boer et al. (2021), and Çimke and Cerit (2021) who found a positive relationship between internet addiction and traditional and cyberbullying. It is somewhat surprising that such a finding was not obtained in the present study. Even though this is highly unlikely, those addicted to the internet in Nigeria may be suffering from a social anxiety disorder or have a dark triad personality that could influence more issues and criminal acts than social bullying (Onyedire et al., 2019). They have come to rely on the internet for all of their day-to-day needs to cope with their inability to interact socially (Lin et al., 2020) and probably enhance their dark personality (Onyedire et al., 2019). As a result, they have developed a strong desire to avoid social situations and everyday life (Omoniyi, 2013). They will not be socially bullied if they cannot maintain a social conversation (Lin et al., 2020).

The result further showed that internet addiction did not moderate the relationship between IGD and social bullying. Thus, the fourth hypothesis was rejected. That is to say, internet addiction neither strengthens the relationship between IGD and social bullying nor lessens it. Even though IGD strengthens social bullying, one would expect that internet addiction would strengthen that relationship further. As such, this seems to be an astonishing finding. However, it could be argued that adolescents and students in Nigeria engage or log in to the internet for more than 6 h a day because of their perceived popularity on social media sites (Omoyemiju and Popoola, 2021). They want to accumulate “likes” and “follows” from Instagram, Facebook, Twitter, YouTube, MySpace, and other related social media posts on the internet (Afolabi et al., 2022). According to the Uses and Gratification Theory, these individuals (victims of the social bully) develop a coping mechanism by using these social media to gratify and divert their attention to the internet, which could be part of a coping strategy to protect themselves from being bullied (Musa et al., 2015). However, social bully perpetrators indulge in social media for more internet or online relationships instead of physical, social bullying of already known friends (Omoyemiju and Popoola, 2021). Adolescents and students use social media and the internet for gratification without any intent to get addicted, disordered, or initiate bullying (Musa et al., 2015).

National Crime Prevention Council [NCPC] (2021), stated that there are three uses and gratification reasons of the internet that might trigger addiction. One, positive suggestion from friends is to use the internet to find lost friends, relatives, and classmates. Two, to become entertained, popular amongst friends and find people with mutual interests. Finally, gather information regarding Students’ course of study and possibly find information regarding on-and-off school and campus events. Or, in today’s world of globalization, students are using the internet to be adequately informed about the trend going on in the world. This might be why our study finding appeared the way it seemed. Also, Nigeria is a heterogeneous country with diverse cultures and religions. These cultures and beliefs might have affected or influenced the uses and gratifications of these adolescents to the point of different perceptions, uses, and experiences of gratification (Musa et al., 2015). As the purpose of using the internet, getting addicted and gratification might result from cultural and religious affiliation. That is to say that the use and gratification theory has different purposes and uses for Nigerian adolescents and students with different cultures and beliefs (Musa et al., 2015). Thus, the reason internet addiction is neutral and could not moderate this relationship.

Finally, the result showed that internet addiction did not moderate the relationship between risk-taking and social bullying among undergraduates. This finding indicates that internet addiction did not influence the direction of the relationship between risk-taking and social bullying and the magnitude of the relationship. That is to say, when a person is already into taking a risk, the introduction of internet addiction contributes nothing to that association (Poon, 2016). Even though this is a novel study, the Use and Gratification Theory can better explain why this played out this way. It is possible that the reason why adolescents and students make use of the internet is for interpersonal utility, such as participating in an educative discussion, making input to burning national issues, belonging to a group without revealing true identity and expressing self freely, learning relevant things that are concerning their academic pursuit and learn more about friends, relatives and classmates (Ozcinar, 2011). Getting involved in these seems to have minimal risks compared to searching for dates, other internet risk-taking behavior and social bullying.

Also, Wireless Fidelity (Wifi) availability in some of their classrooms and hostels may have highlighted the convenience associated with internet addiction. Availability must have made the internet venue very convenient and thus, communication with significant others very cheap and attractive. Although availability and convenience may trigger internet risk-taking behaviors, such as internet dating, pornography viewing preference, gaming, sexual abuse and addiction, incessant strikes by Nigerian university lecturers have made these impossible since most semesters are shorter than expected, thus less time for frivolities. Therefore, since the perceived use of Use and Gratification Theories differs according to perception, experiences, cultures, and religions, it is difficult for internet addiction to moderate the relationship between risk-taking and social bullying in a heterogeneous country like Nigeria.

### Limitations

Our study, however, has some limitations. The small sample size of 552 and the study location (South-East Nigeria) appear to limit the generalizability of this conclusion. Future research samples should be more extensive, and the scope of data collection should be increased to encompass more sections of Nigeria. In addition, there seem to be more females than males in Nigerian universities. Possibly, this is a significant limitation that could influence the result. Future studies need to address this issue. Also, a cross-sectional study cannot infer causality between variables. Our findings showed that internet addiction did have no significant relationship with social bullying. Future research on this topic has a compelling case to be made.

Furthermore, the study design can be improved by including other health-related and psychological variables. As a result, it can be used to investigate further and back up the current study’s findings, which found no link between internet addiction and social bullying. In future studies, using a longitudinal technique to determine the etiology of an effect could be beneficial.

### Suggestions for Further Studies

Parents, school administrators, and others should investigate and study positive family, school, and other significant climates in which undergraduates assemble. Internet addiction and gambling disorder, risk-taking, and social bullying can be classified for proper diagnosis and treatment. Furthermore, these essential people in Students’ lives should teach them empathy and kindness. Those who possess these qualities are less likely not to get addicted to the internet, develop IGD, engage in risky behavior, or engage in social bullying. Parents, school authorities, and significant others must recognize gateway behaviors and warning signs in these undergraduates by providing opportunities for them through interactions. Helping these students connect and feel that their friends and significant others are a part of their group or community reduces the likelihood of bullies bullying their victims. While many onlookers want to protect bullies from their victims, groups and communities are ripping apart the society they fought so hard to build.

In the same way, children should be taught to use the arts as a powerful instrument for reflecting, expressing, and engaging in stimulation classes and connecting with instructors, parents, and significant people about their experiences of social bullying, IGD, risk-taking behavior, and internet addiction. Furthermore, these significant others can bring peaceful solutions and answers to social bullying, internet gaming behavior, and risk-taking behavior by removing labels, addressing behaviors, setting clear, enforceable rules, using open communication, monitoring students hot spots, acknowledging the uniqueness of each child by lending a listening ear, and rewarding positive behavior for these students. Furthermore, these significant others may help these pupils by thinking that they will thrive if they take care of them, participate in activities, and track their progress. Anti-bullying programs can now be tailored to specific individuals to match various groups of people better.

## Conclusion

Even if the violence is not limited to school grounds, education plays a critical role in teaching students to navigate the digital world appropriately. To protect themselves and others from cyberbullying, IGD, online risk-taking behaviors, and addiction. Children and adolescents should be taught how to behave civilly online, cope with stress, identify online aggression, and report it to the appropriate authorities, all of which should be taught in school.

Every country has a problem with social bullying in schools and universities. Bullying has a wide range of negative consequences for children, and confronting and resolving social bullying situations among adolescents requires adult involvement and commitment. According to the poll, male university students were more likely than female university students to engage in physical and verbal bullying, with the latter being the most common form of bullying among female students. According to survey respondents, bullying has also been linked to stress, unemployment, social status, jealousy, teasing, and retribution.

Finally, IGD and risk-taking behavior were positive predictors of social bullying, although internet addiction neither predicted nor moderated the association. IGDs and risk-taking behavior have increased the likelihood of college students being bullied by others.

## Data Availability Statement

The raw data supporting the conclusions of this article will be made available by the authors, without undue reservation.

## Ethics Statement

Written informed consent was obtained from the individual(s), and minor(s) legal guardian/next of kin, for the publication of any potentially identifiable images or data included in this article.

## Author Contributions

CN and IU contributed significantly to the writing of the manuscript and analysed and interpreted the data. GK designed the study, who also came up with the hypothesis. GK, OI, and TO aided in the study’s planning, collection of data, and design. IU, GK, TO, and OI critically revised the article for intellectual substance. CN, IU, OI, and TO wrote the literature review and discussion. All authors offered critical comments and assisted in developing the study, analysis, and text, and have given their approval to the final version.

## Conflict of Interest

The authors declare that the research was conducted in the absence of any commercial or financial relationships that could be construed as a potential conflict of interest.

## Publisher’s Note

All claims expressed in this article are solely those of the authors and do not necessarily represent those of their affiliated organizations, or those of the publisher, the editors and the reviewers. Any product that may be evaluated in this article, or claim that may be made by its manufacturer, is not guaranteed or endorsed by the publisher.
